# Supplementary material for: The uterine secretome initiates growth of gynecologic tissues in ectopic locations
Source: PLoS One. 2024 May 10;19(5):e0292978. doi: 10.1371/journal.pone.0292978 (PMC11086859; doi:10.1371/journal.pone.0292978)
Supplement: Attachment — Submitted filename: 20231219 response to reviewers.docx [file pone.0292978.s003.docx]

We apologize for the failure of the citations to update properly, these changes had been made however our reference manager software added them back which was an oversight on our part. We have now corrected and confirmed these changes

The updated citations are as follows in the final version (line numbers from the tracked changes version):

579 2. Sunde J, Wasickanin M, Katz TA, Wickersham EL, Steed DOE, Simper N. Prevalence of endosalpingiosis and other benign gynecologic lesions. PloS one. 2020;15(5):e0232487. doi: 10.1371/journal.pone.0232487. PubMed PMID: 32401810

655 25. Somigliana E, Vigano P, Zingrillo B, Ranieri S, Filardo P, Candiani M, et al. Induction of endometriosis in the mouse inhibits spleen leukocyte function. Acta obstetric et gynecolog Scand. 2001;80(3):200-5. doi: 10.1034/j.1600-0412.2001.080003200.x. PubMed PMID: 11207484.

690 37. Grasso A, Navarro R, Balaguer N, Moreno I, Alama P, Jimenez J, et al. Endometrial Liquid Biopsy Provides a miRNA Roadmap of the Secretory Phase of the Human Endometrium. J clin endocrinolmetab. 2020;105(3). doi: 10.1210/clinem/dgz146. PubMed PMID: 31665361.

734 50. Steenbeek MP, van Bommel MHD, Bulten J, Hulsmann JA, Bogaerts J, Garcia C, et al. Risk of Peritoneal Carcinomatosis After Risk-Reducing Salpingo-Oophorectomy: A Systematic Review and Individual Patient Data Meta-Analysis. J Clin Oncol. 2022;40(17):1879-91. doi: 10.1200/JCO.21.02016. PubMed PMID: 35302882

745 52. Hanley GE, Pearce CL, Talhouk A, Kwon JS, Finlayson SJ, McAlpine JN, et al. Outcomes From Opportunistic Salpingectomy for Ovarian Cancer Prevention. JAMA Netw Open. 2022;5(2):e2147343. doi: 10.1001/jamanetworkopen.2021.47343. PubMed PMID: 35138400

Thank you again for the opportunity to share our work.

Prior revisions:

We would like to thank the editor and the reviewers for the comments on our manuscript we have added a more in-depth discussion on endometriosis (lines 497-552). The comments from reviewers are addressed in a point-by-point manner with our responses in red below

Reviewer #1: In this study, Sunde et al., propose a new mouse model to study endometriosis pathogenesis and give some data supporting a causative effect for the uterine secretome. The study is straightforward and might be interesting for the scientific community. I just have some minor comments:

- The English is understandable but needs some improvement in grammar and syntax for clarity.

Thank you for this comment we have gone through the manuscript and improved the writing throughout

- Usually, manuscripts submitted for peer review are given with numbered lines; this helps both the work of the reviewers and authors.

Thank you for this comment and we apologize for the oversight on our first submission– line numbers have been added

- Please add the reference to this sentence: “Specified genes have been demonstrated to play a role in the pathogenesis of ovarian inclusion cysts in transgenic models, but these models may also unexpectedly alter genes normally involved in lesion development and these models are not designed to evaluate the role of uterine secretome factors in ectopic lesion development.”

References have been added (line 84)

- In all histological figures, the magnification used, and the reference size are missing, even though some are reported in the Fig legends. Please provide this information on the image.

Thank you for this comment, scale bars have been added to all images

- In Fig. 1B, please specify to which implantation the images refer to.

Thank you for this comment, arrows have been added to figure 1B to point out the implantation sites we are referring to (line 295)

- Fig.2A is not mentioned in the text. Please describe Fig.2A before introducing Fig.2B in the text.

Thank you for this comment, we have added reference to figure 2A in the results section (line 316)

- Fig. 3 is very poorly described; please complete it with additional information.

Thank you for this comment, both figure 3 as well as the figure 3 legends have been updated (lines 337-338)

- I suggest fusing the paragraph titled “Hormonal cycling in both donor and recipient mice affect lesion initiation” and the paragraph titled “Leukemia inhibitory factor improves lesion implantation of oviductal tissue” in one.

Thank you for this comment, we have combined these sections under the new heading of “Leukemia inhibitory factor and hormonal cycling impact lesion implantation and initiation” line 377

Reviewer #2: The article is well written and the data are novel and original. I have only few comments
1) The quality of the histological picture shown in figure 3 is poor. I suggest the authors to improve them, because it is difficult for the reader to analyze them

Thank you for this comment – we have updated figure 3 to include more clear and larger images

2) Pathogenesis of endometriosis is still not completelty defined and there are other mechanisms proposed more than the Sampson theory and the free floating gynecologic epithelial cells. It would be interesting to discuss the data produced respect to other pathogenetic mechanisms proposed for endometriosis such as celomic metaplasia or embryologic theory

Thank you for this comment, we have added more discussion on endometriosis lines 499-554

Reviewer #3: Dear Authors
Thank you for your valuable submission. I read your with the utmost interest and pleasure, however the reading was demanding and required a deep immersion in the field.

I found a small letter mistake (mataplasia instead of metaplasia) in the results chapter

Thank you for this comment this has been fixed (line 422)

and discrepancies in bibliography, where
some additional text was put in: pos 2, 50, 52
full name of the journal instead of the abbreviation: pos 25, 37

Thank you for these comments – we have fixed these citation errors

Nevertheless, congratulations for your work. It is my pleasure to recommend it for publication.

Thank you for your kind comments

Journal Requirements:

When submitting your revision, we need you to address these additional requirements.

1. Please ensure that your manuscript meets PLOS ONE's style requirements, including those for file naming. The PLOS ONE style templates can be found at
<https://journals.plos.org/plosone/s/file?id=wjVg/PLOSOne_formatting_sample_main_body.pdf> and
<https://journals.plos.org/plosone/s/file?id=ba62/PLOSOne_formatting_sample_title_authors_affiliations.pdf>

We have verified the formatting meets PLOS ONE’s style requirements

2. We note that you have stated that you will provide repository information for your data at acceptance. Should your manuscript be accepted for publication, we will hold it until you provide the relevant accession numbers or DOIs necessary to access your data. If you wish to make changes to your Data Availability statement, please describe these changes in your cover letter and we will update your Data Availability statement to reflect the information you provide.

There is no data to deposit in a data repository, all raw data are provided in the data repository tables

3. Please upload a copy of Figure 7, to which you refer in your text on page 26. If the figure is no longer to be included as part of the submission please remove all reference to it within the text.

Thank you for this comment, this figure reference should have read figure 4 – it has been corrected

4. Please include captions for your Supporting Information files at the end of your manuscript, and update any in-text citations to match accordingly. Please see our Supporting Information guidelines for more information: <http://journals.plos.org/plosone/s/supporting-information>.

A caption for the supporting data availability files has been added

5. Please review your reference list to ensure that it is complete and correct. If you have cited papers that have been retracted, please include the rationale for doing so in the manuscript text, or remove these references and replace them with relevant current references. Any changes to the reference list should be mentioned in the rebuttal letter that accompanies your revised manuscript. If you need to cite a retracted article, indicate the article’s retracted status in the References list and also include a citation and full reference for the retraction notice.

We have reviewed all our references for correctness and to make sure none of them have been retracted
